# The nitridation of ZnO nanowires

**DOI:** 10.1186/1556-276X-7-175

**Published:** 2012-03-08

**Authors:** Matthew Zervos, Chrystalla Karipi, Andreas Othonos

**Affiliations:** 1 Nanostructured Materials and Devices Laboratory, Department of Mechanical Engineering, Materials Science Group, University of Cyprus, P.O. Box 20537, Nicosia, 1678, Cyprus; 2 Research Center of Ultrafast Science, Department of Physics, School of Physical Sciences, University of Cyprus, P.O. Box 20537, Nicosia, 1678, Cyprus

**Keywords:** zinc oxide, zinc nitride, nanowires, nitridation

## Abstract

ZnO nanowires (NWs) with diameters of 50 to 250 nm and lengths of several micrometres have been grown by reactive vapour transport via the reaction of Zn with oxygen on 1 nm Au/Si(001) at 550°C under an inert flow of Ar. These exhibited clear peaks in the X-ray diffraction corresponding to the hexagonal wurtzite crystal structure of ZnO and a photoluminescence spectrum with a peak at 3.3 eV corresponding to band edge emission close to 3.2 eV determined from the abrupt onset in the absorption-transmission through ZnO NWs grown on 0.5 nm Au/quartz. We find that the post growth nitridation of ZnO NWs under a steady flow of NH_3 _at temperatures ≤600°C promotes the formation of a ZnO/Zn_3_N_2 _core-shell structure as suggested by the suppression of the peaks related to ZnO and the emergence of new ones corresponding to the cubic crystal structure of Zn_3_N_2 _while maintaining their integrity. Higher temperatures lead to the complete elimination of the ZnO NWs. We discuss the effect of nitridation time, flow of NH_3_, ramp rate and hydrogen on the conversion and propose a mechanism for the nitridation.

## Introduction

Semiconductor nanowires (NWs) have been investigated intensively over the past decade in view of their potential as emerging materials and devices for the extension of Moore's law and the upsurging interest in nanotechnology. Metal-oxide (MO) semiconductor NWs like ZnO [1], In_2_O_3 _[2], Ga_2_O_3 _[3] and SnO_2 _[4] have been studied, and ZnO NWs in particular have been used for the fabrication of novel nanoscale devices such as light-emitting diodes [5], lasers [6], solar cells [7] and sensors [8]. On the other hand, III-V compound semiconductors and in particular nitrides such as InN [9] and GaN NWs [10-12] have also been investigated in view of their technological applications, e.g. solar cells, especially as it is possible to tune their energy band gap over a wide range like for instance in InGaN [13]. Recently, we also demonstrated the conversion of In_2_O_3 _[14] and Ga_2_O_3 _NWs [15] into their respective nitrides, i.e. InN and GaN, using different post growth nitridation strategies including NH_3 _and H_2_.

Despite intensive investigations on ZnO NWs which have an energy band gap of *E*_G _= 3.3 eV very little is known about Zn_3_N_2 _NWs, while the nitridation and conversion of ZnO into Zn_3_N_2 _NWs has not been studied at all. More specifically, Zn_3_N_2 _NWs have only been obtained by Zong et al. [16] via the direct reaction of Zn with 250 sccm of NH_3 _at 600°C. The Zn_3_N_2 _NWs had diameters ≈100 nm and lengths between 10 and 20 μm but their diameters were not uniform and were grown within the Zn powder itself, not on a substrate. Irregular, Zn_3_N_2 _hollow-like spheres with diameters of ≈3 μm were also obtained under identical growth conditions [17]. However, there are no other studies on nanostructured Zn_3_N_2_. On the other hand, nitrogen doping of ZnO NWs is a topic of active interest since nitrogen is considered to be a shallow-like, p-type impurity in ZnO [18] although recently, it was claimed to be a deep acceptor not capable of giving p-type ZnO [19]. However, very small flows, e.g. 0.2 sccm of NH_3_, have been used together with O_2 _during the growth of such ZnO NWs [20], and no changes occur in the crystal structure by post growth nitrogen doping [21].

Therefore, we have undertaken a systematic investigation into the post growth nitridation and conversion of ZnO NWs into Zn_3_N_2 _NWs, thereby complementing our earlier investigations on the nitridation of other MO NWs [14,15]. ZnO NWs with diameters of 50 to 250 nm, lengths up to 10 μm and a hexagonal wurtzite crystal structure were obtained with a high yield and uniformity not only on ≈1 nm Au/Si(001) and 1 nm Au/quartz via reactive vapour transport, but also on plain Si(001) at 550°C. We find that the post growth nitridation of ZnO NWs under a steady flow of NH_3 _is feasible for temperatures ≤600°C since they maintain their integrity, and we observe not only a suppression of the X-ray diffraction (XRD) peaks related to ZnO, but also the emergence of new ones corresponding to the cubic crystal structure of Zn_3_N_2 _identical to that of Zn_3_N_2 _layers which were grown on Au/Si(001) via the direct reaction of Zn with NH_3_. Temperatures higher than 600°C lead to the complete elimination of the ZnO NWs. We describe how the temperature-time profile and flows of Ar, NH_3 _and H_2 _influence the conversion of ZnO into Zn_3_N_2 _and propose a mechanism whereby N reacts with Zn at the surface of the ZnO NWs undergoing reduction due to hydrogen and leading to the formation of a core-shell structure.

## Experimental methods

ZnO NWs were grown using an atmospheric pressure chemical vapour deposition reactor consisting of four mass flow controllers and a 25-mm horizontal quartz tube (QT) furnace capable of reaching 1,100°C. For the growth of ZnO NWs, Zn pellets (2-14 mesh, 99.9%, Aldrich, Sigma-Aldrich Corporation, St. Louis, MO, USA) were cut into small fragments that were weighed individually with an accuracy of ± 1 mg. Square pieces of p^+^Si(001) ≈7 mm × 7 mm or quartz were cleaned sequentially in methanol, acetone and isopropanol; rinsed with deionised water; dried with N_2 _and coated with Au having a thickness of ≈0.5 to 20 nm by sputtering using Ar at 1 × 10^-2 ^mbar.

After carefully loading 0.2 to 1.0 g of Zn fragments and the Au/p^+^Si(001) substrates into a clean 100-mm-long, combustion-type boat and recording carefully their positions and relative distances, the boat was inserted in the QT which was subsequently purged with a flow of 500 sccm of Ar for 5 min, and then the temperature was ramped to the desired growth temperature (*T*_G_) using a ramp rate of 30°C/min and 100 sccm of Ar. Upon reaching *T*_G_, the same flow of Ar was maintained for a further 60 min, after which the reactor was allowed to cool down slowly for at least 30 min without changing the flow of Ar. The sample was always removed when the temperature was lower than 100°C, and the weight of the remaining Zn was measured to find the amount transferred into the main gas stream. The QT was changed regularly in order to maintain a clean, high-temperature zone for the growth of the ZnO NWs. Low-pressure reactive vapour transport was carried out by connecting a chemically resistant, two-stage rotary pump, downstream for the purpose of controlling the partial pressures of O_2 _and Ar which were supplied to the QT via a micro-flow leak valve connected after the mass flow controller manifold on the upstream side. A pressure gauge was connected upstream after the micro-flow leak valve in order to monitor the pressure. The morphology of the ZnO NWs was examined with a TESCAN scanning electron microscope (SEM) (Brno, Czech Republic), while their crystal structure and phase purity were determined using a SHIMADZU, XRD-6000, X-ray diffractometer with Cu-Kα source (Kyoto, Japan), by performing a scan of *θ*-2*θ *in the range between 10° and 80°. Furthermore, the photoluminescence (PL) of the ZnO NWs grown on Au/Si(001) was also measured at 300 K using excitation at *λ *= 267 nm, while the absorption-transmission spectra of the ZnO NWs grown on Au/quartz were measured with a PerkinElmer UV-Vis spectrophotometer (Waltham, MA, USA). No PL was carried out on the ZnO NWs grown on Au/quartz since the latter emits light under excitation at *λ *= 267 nm in contrast to the case of Si. For the nitridations, as-grown ZnO NWs were loaded in a boat without any solids which was positioned over the thermocouple as described above. Then, the QT was purged with a flow of 500 sccm of Ar or N_2_:10% H_2 _for 10 min, and the temperature was ramped to the desired nitridation temperature (*T*_N_) using different ramp rates of 10°C/min to 30°C/min under a flow of 250 sccm of Ar or N_2_:10% H_2_. Upon reaching *T*_N_, the flow of Ar was interrupted and 50 to 500 sccm of NH_3 _was introduced for 60 to 120 min, after which the reactor was allowed to cool down slowly for at least 30 min without changing the flow of NH_3_. The changes in morphology, crystal structure and optical properties were determined again using the methods outlined above.

## Results and discussion

**Figure 1:**
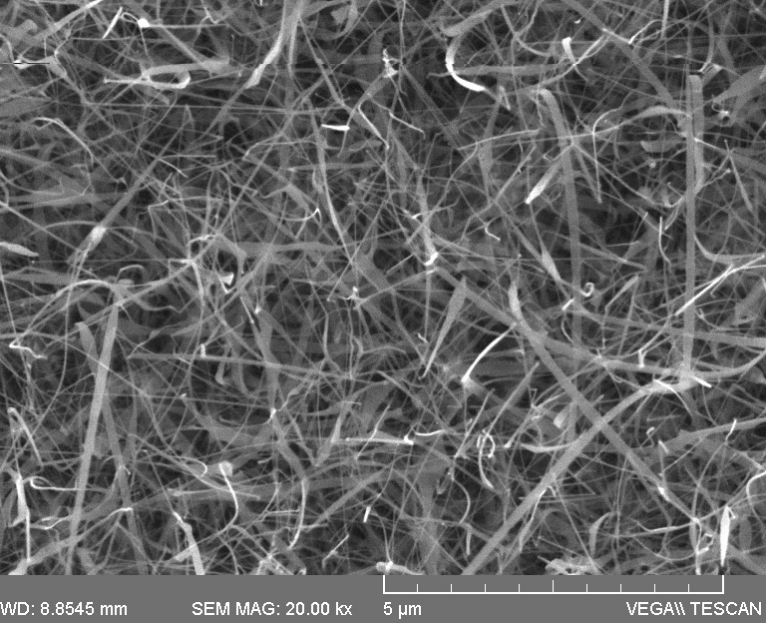
**Typical image of ZnO NWs grown on 1.0 nm Au/quartz at 550°C**.

**Figure 2:**
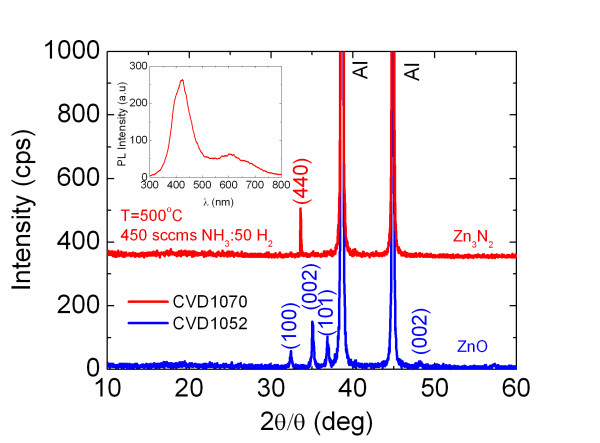
**XRD spectra**. ZnO NWs grown on 1.0 nm Au/Si(001), lower trace and Zn_3_N_2 _layer grown on Au/Si(001) using H_2_, top trace. Inset shows the corresponding RT PL of Zn_3_N_2_. The peaks belonging to the Al holder have also been identified and labelled.

To date, a great variety of ZnO nanostructures such as nanowires, nanobelts, nanotubes, nanohelices and nanorings have been grown, and their properties have been investigated accordingly [1]. In particular, ZnO NWs have been grown by various methods such as reactive vapour transport using carbo-thermal reduction of ZnO at 900°C but also via the direct reaction of Zn with O_2 _[22,23]. The latter method avoids the incorporation of C in ZnO and the use of high temperatures for the purposes of reduction. We begin by describing the growth of ZnO NWs. A typical SEM image of ZnO NWs obtained at 550°C on 1 nm Au/Si(001) is shown in Figure 1. A high-yield, uniform distribution of ZnO NWs having diameters of 50 to 250 nm and lengths of a few micrometres was obtained which exhibited clear peaks in the XRD as shown in Figure 2 corresponding to the hexagonal wurtzite crystal structure of ZnO [24]. No deposition occurred at temperatures < 500°C since Zn has a melting point of 420°C. ZnO NWs were obtained at 500°C, 550°C and 600°C due to the higher vapour pressure of Zn which is ≈14 mBar at 600°C [16]. We find that the amount of Zn transferred into the gas stream increased with temperature. However, higher temperatures, i.e. 700°C, 800°C and 900°C, were not favourable towards the growth of ZnO NWs despite the complete transfer of Zn into the gas stream and even higher metal vapour pressure. Instead, the reaction of Zn with O_2 _leads to the preferential deposition of a layer.

ZnO NWs were grown optimally with a high yield and uniformity at 550°C under a steady flow of 100 sccm of Ar on 1.0 nm Au/Si(001) which was positioned ≈5 to 10 mm from 0.2 g of Zn fragments. The ZnO NWs appeared as a white blue-like deposit after the reaction, but the yield decreased to zero at a distance of a few tens of millimetres. Increasing the flow of Ar up to 500 sccm leads to a higher yield over distance and a reduction in the percentage of O_2 _in the reactor. In contrast, the addition of 20% O_2_, i.e. 20 sccm of O_2 _and 80 sccm of Ar at 1 atm, leads to a complete suppression in the growth of ZnO NWs, keeping everything else equal at optimum conditions. No deposition occurred at all on the Au/Si(001) surface which maintained its mirror-like appearance since the vapour pressure of Zn was effectively reduced to zero as no metal was transferred into the main gas stream. This is due to the oxidation of the source of Zn fragments which is a well-known drawback in the synthesis of MO NWs by the reactive vapour transport method where the metal constituent is included *in situ *. In fact, the vapour pressure of Zn was also zero at 10^-1 ^mbar using 98% Ar:2% O_2 _mixture at 500°C for the same reason. In addition to changing the temperature, we varied the mass of Zn used in the reaction between 0.2 and 1.5 g at 550°C using 100 sccm of Ar which gave Zn-rich conditions but did not obtain a high yield of ZnO NWs. Therefore, the growth of ZnO NWs depends critically on (a) the vapour pressure of Zn which in turn depends on the temperature and the mass of Zn, (b) partial pressure of O_2 _and (c) catalyst size and type of substrate, in decreasing order of importance.

As far as the latter is concerned, we find that Au promotes the growth of ZnO NWs on Si(001), but it is not indispensible as such and in fact hindered the growth of ZnO NWs when we used > 10 nm Au/Si(001) at 550°C, most likely due to a suppression in the formation of Au NPs. ZnO NWs were also obtained on plain Si(001) under optimum conditions at 550°C, suggesting that they grow via a self-catalysed vapour-solid mechanism similar to ZnO NWs which have been obtained by post growth oxidation of Zn layers deposited on Si(001) [22]. However, ZnO NWs did not grow on plain quartz but only on Au/quartz as shown in Figure 1, suggesting that the type of substrate is also important.

**Figure 3:**
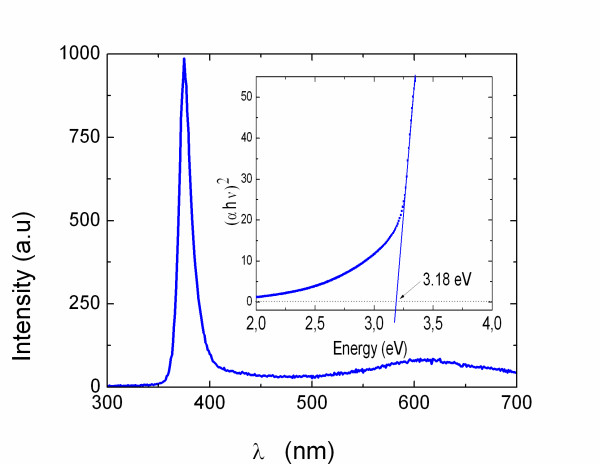
**Typical RT PL of ZnO NWs grown on 1.0 nm Au/Si(001) at 550°C**. The inset shows a plot of *α*^2 ^versus energy from ZnO NWs grown on 0.5 nm Au/quartz.

The room-temperature (RT) PL of the ZnO NWs obtained under optimum conditions at 550°C is shown in Figure 3 where the peak at 3.3 eV (≡375 nm) corresponds to band edge emission in agreement with the energy band gap of ZnO. The residual emission at 600 nm is attributed to radiative recombination via states that are energetically located within the energy band gap and which are related to structural defects and surface states [2-4]. The square of the absorption versus wavelength through the ZnO NWs grown on 0.5 nm Au/quartz is shown as an inset in Figure 3 from which one may observe an abrupt reduction in the absorption at 3.2 eV close to the energy band gap of ZnO in very good agreement with the PL.

In addition to ZnO NWs, we attempted to grow Zn_3_N_2 _NWs on Au/Si(001) via reactive vapour transport and the direct reaction of Zn with NH_3_. No Zn_3_N_2 _NWs were obtained by varying the temperature between 500°C and 700°C, flow of NH_3 _or amount of Zn. Instead, we always obtained uniform layers with a characteristic blue- or yellow-like colour while no deposition occurred on plain Si(001). In particular, the reaction of Zn with 450 sccm of NH_3 _containing 50 sccm of H_2 _over 1 nm Au/Si(001) gave a uniform layer which exhibited a single peak at *θ *= 33.3° in the XRD, as shown in the inset of Figure 2, corresponding to the (440) crystallographic direction and cubic crystal structure of Zn_3_N_2_. The corresponding RT PL is shown as an inset in Figure 2 with a maximum at *λ *= 422 nm, red shifted by ≈50 nm with respect to the ZnO PL shown in Figure 3.

**Figure 4:**
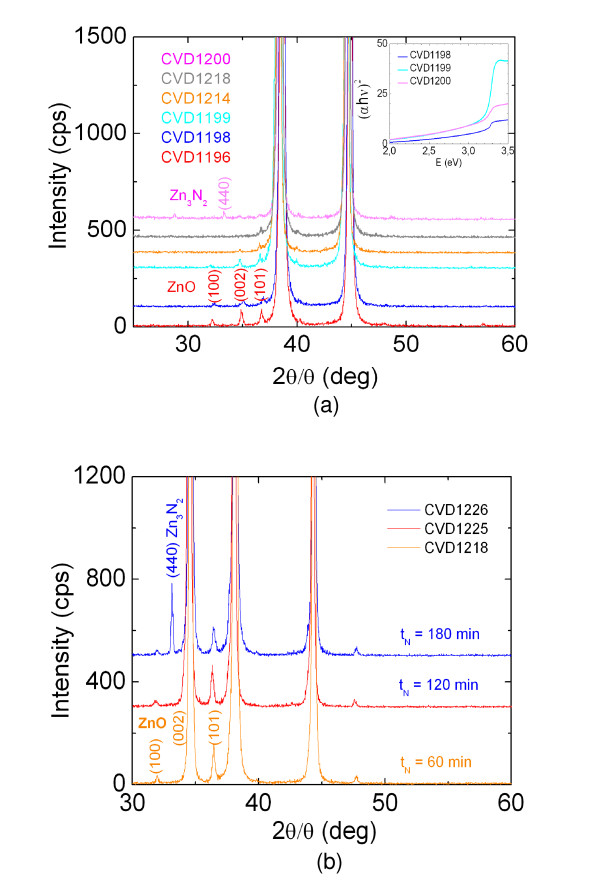
**XRD spectra of treated ZnO NWs**. (**a**) ZnO NWs which were treated under NH_3 _at various temperatures, flows of NH_3_, nitridation times and ramp rates arranged in the same order as Table 1. (**b**) ZnO NWs which were treated repeatedly under 250 sccm of NH_3 _at 600°C for 60 min; see Table 1.

Following the growth of ZnO NWs, we carried out post growth nitridations between *T*_N _= 500°C and 700°C for 1 h using 250 sccm of NH_3 _as listed in Table 1 (CVD1194-1198). In this case, the temperature was increased to *T*_N _at 30°C/min under an inert gas flow of 250 sccm of Ar, after which NH_3 _was admitted into the reactor. We find that the ZnO NWs are completely eliminated for temperatures > 600°C. This is similar to the case of other MO NWs such as In_2_O_3 _[2] and SnO_2 _[4] and is attributed to the rapid reduction of ZnO under NH_3 _which occurs at high temperatures due to hydrogen evolving from NH_3 _and leads to the elimination of the ZnO NWs. Temperatures ≤600°C did not lead to any significant changes in the crystal structure of the ZnO NWs using Ar during the ramp and small to moderate flows of 50 to 250 sccm of NH_3 _for 60 min (CVD1199) as shown in Figure 4a since we still observe the (101), (002) and (101) peaks of ZnO which are shown in Figure 2.

**Table 1:** Summary of post growth nitridation conditions used for the conversion of ZnO into Zn_3_N_2 _NWs

	*T*_N_ (°C)	NH_3_ (sccm)	*t*_N_ (min)	Ramp rate (°C/min)
CVD1196	500	250	60	30
CVD1198	550	250	60	30
CVD1194	600	250	60	30
CVD1197	630	250	60	30
CVD1195	700	250	60	30
CVD1199	600	50	60	30
CVD1217	600	500	60	30
CVD1214	600	250	90	30
CVD1218	600	250	120	30
CVD1200	600	250	60	10
CVD1211	600	500	60	10

*T*_N_, nitridation temperature; *t*_N_, nitridation time.

However, larger flows of 500 sccm of NH_3 _(CVD1217) inadvertently lead to the elimination of the ZnO NWs. Similarly, the use of 250 sccm of N_2_:10% H_2 _as opposed to Ar during the temperature ramp leads to the complete elimination of the ZnO NWs after performing nitridations at 600°C under 250 sccm of NH_3 _for 60 min.

In contrast, we find that there is a suppression and gradual elimination of the peaks related to ZnO NWs by increasing the nitridation time from 60 min up to 90 min under a flow of 250 sccm of NH_3 _as listed in Table 1 (CVD1214) and shown in Figure 4a. This was clearly evident upon performing another nitridation on the latter under 250 sccm of NH_3 _for a further 60 min (CVD1218) which leaves a single, weak, but nevertheless clearly resolved peak at *θ *= 36.5°.

The ZnO NWs seem to be converted into Zn_3_N_2 _NWs using lower ramp rates of 10°C/min and 250 sccm of NH_3 _(CVD1200) throughout the entire process as opposed to Ar during the temperature ramp since there is a more or less complete suppression of the peaks related to ZnO NWs as shown in Figure 4a, and a single, new peak corresponding to the (440) crystallographic direction and cubic crystal structure of Zn_3_N_2 _[25] has appeared while the nanowires maintain their integrity. Using a higher flow rate of 500 sccm of NH_3 _(CVD1211) or by extending the nitridation time to 90 min and using NH_3 _throughout the entire nitridation process leads to a significant reduction and complete elimination of the ZnO NWs.

Here, a lower ramp rate is essentially equivalent to a longer nitridation time, while a higher flow of NH_3 _is equivalent to increasing the hydrogen which is detrimental to the ZnO NWs. A plausible mechanism for the nitridation process which explains the above findings is as follows: during the nitridation process, hydrogen evolving from NH_3 _leads to a reduction of ZnO [26] and Zn reacts with N at the surface of the ZnO NWs, forming a ZnO/Zn_3_N_2 _core-shell structure. Increasing the nitridation time simply allows nitrogen to diffuse into and oxygen out of the core which is reflected in the gradual suppression of the XRD peaks related to ZnO NWs which maintain their integrity. Temperatures higher than critical and/or excess hydrogen inadvertently lead to a rapid reduction of the ZnO NWs and their elimination.

It is worthwhile pointing out that the ZnO NWs were also eliminated by performing an extended two-step nitridation: first at 500°C for 60 min and then at 620°C for another 60 min using a low ramp rate of 10°C/min and a steady flow of 250 sccm of NH_3 _throughout the entire process. Therefore, the nitridation temperature is highly critical and should not exceed *T*_N _= 600°C. Another significant aspect in the conversion of ZnO to Zn_3_N_2 _NWs is that their diameters are of the order of a few tens of nanometres, i.e. as thin as possible.

We find that thick ZnO NWs and the underlying layer exhibit intense oxide peaks in the XRD even after performing repeated nitridations at *T*_N _= 600°C under 250 sccm of NH_3 _and using 250 sccm of Ar during the temperature ramp as shown in Figure 4b. However, we clearly observe the appearance of the (440) peak of Zn_3_N_2 _after repeated nitridations of 180 min, consistent with that shown in Figures 2 and 4a.

From the above, it appears that in most cases, we have the formation of a ZnO/Zn_3_N_2 _core-shell structure. However, it is important to mention that the (100), (002) and (101) peaks of the ZnO NWs shown above are very similar to the (222), (321) and (400) peaks of the Zn_3_N_2 _layers prepared by Futsuhara et al. [27], the Zn_3_N_2 _NWs of Zong et al. [16,17] and the Zn_3_N_2 _powders of Partin et al. [25]. Therefore, further investigations on the composition and structural properties are required in order to determine if there is a total conversion from ZnO into Zn_3_N_2_.

Finally, the absorption-transmission spectra of the ZnO NWs grown on quartz which were treated under NH_3 _exhibit an energy band gap around 3.2 eV as shown in the inset of Figure 4a which is consistent with the energy band gap of the Zn_3_N_2 _NWs of Zong et al. [16] who used similar growth conditions and which exhibited RT PL at 3.2 eV but also consistent with the PL of the Zn_3_N_2 _layer shown in Figure 2. While no PL was carried out on the ZnO/Zn_3_N_2 _core-shell NWs grown on Au/quartz, it is very likely that the formation of the Zn_3_N_2 _shell will result into the separation of photoexcited electron-hole pairs and therefore change the radiative recombination rates due to a reduced wave function overlap especially if we have the formation of a core-shell p-n junction and a related electric field.

## Conclusion

ZnO NWs with diameters of 50 to 250 nm, lengths up to 10 μm and a hexagonal wurtzite crystal structure have been grown on 1 nm Au/Si(001) or Au/quartz at 550°C under an inert gas flow of Ar. These exhibited an energy band gap of *E*_G _= 3.2 to 3.3 eV determined from photoluminescence and absorption transmission steady state spectroscopy. In contrast, the direct reaction of Zn with NH_3 _leads to the preferential growth of Zn_3_N_2 _layers highly oriented along (440) and with a cubic crystal structure on Au/Si(001) but no Zn_3_N_2 _NWs as such.

We find that the post growth nitridation of ZnO NWs under a steady flow of NH_3 _is feasible for temperatures ≤600°C, but in most cases, we have the formation of a ZnO/Zn_3_N_2 _core-shell structure using low ramp rates and moderate flows of 250 sccm of NH_3_. Higher temperatures > 600°C or excessive NH_3 _and H_2 _lead to a reduction and complete elimination of the ZnO NWs. A plausible mechanism is put forward whereby the nitridation leads to the formation of a ZnO/Zn_3_N_2 _core-shell structure and proceeds via the inward diffusion of nitrogen.

## Abbreviations

MO: metal-oxide; NW: nanowire; PL: photoluminescence; QT: quartz tube; SEM: scanning electron microscope; XRD: X-ray diffraction.

## Competing interests

The authors declare that they have no competing interests.

## Authors' contributions

MZ and CK were responsible for the growth and structural characterization of the nanowires. AO was responsible for the measurements of the optical properties. All authors have read and approved the final manuscript.
